# Preliminary investigation and application of a modified objects memory test in perioperative cognitive evaluation

**DOI:** 10.3389/fnbeh.2023.1042836

**Published:** 2023-03-23

**Authors:** Lanfeng Chen, Baobin Gao, Chaoyang Yan, Zhengzheng Wang, Yiqing Bi, Hongfu Chen, Haojie Jin

**Affiliations:** Department of Anesthesiology, Zhoushan Maternal and Child Health Hospital, Zhoushan, China

**Keywords:** verbal learning test, Syndrom Kurztest, postoperative cognitive dysfunction, elderly, neuropsychological test

## Abstract

**Objective:**

To investigate the applicability of a modified verbal learning test redesigned from the memory subtest of the Syndrom Kurztest (SKT) in perioperative cognitive evaluation.

**Methods:**

Patients receiving elective herniorrhaphy and their accompanying family members (set as normal controls), 55–75 years old, were randomly divided into two groups. The two groups received the self-made objects memory test derived from the SKT (SMOT) SMOT or a traditional auditory verbal learning test (AVLT). The cognitive evaluation was administered at the bedside on the day before surgery and the second day after surgery.

**Results:**

The SMOT test was administered to 121 subjects, while 107 patients received the AVLT test. After confirming that there was no significant difference in cognitive function between patients and their family members, the results of the SMOT and AVLT tests were compared. The results showed that the “low-score” ratio of the SMOT was significantly lower than that of the AVLT test (*P* < 0.05), and the influencing factors of the SMOT were less than those of the AVLT test. However, the learning effect of the SMOT was more significant (*P* < 0.05).

**Conclusion:**

This study preliminarily confirms that the SMOT has better applicability to elderly Chinese individuals than AVLT in perioperative cognitive evaluation, but its learning effect should be noted.

## 1. Introduction

Postoperative cognitive dysfunction (POCD) occurs frequently in elderly patients undergoing major surgery. The assessment and diagnosis of POCD require the use of a combination of neuropsychological tests (Hanning, 2005; Evered and Silbert, 2018). The Auditory Verbal Learning Test (AVLT) is one of the classical neuropsychological tests used to assess learning and memory and is widely used for cognitive function assessment (Moller et al., 1998; Rasmussen et al., 2005). Different versions of AVLT have also been widely used in POCD studies, including the International Study Group of Postoperative Cognitive Dysfunction (ISPOCD) (Guo et al., 2007; Zhao et al., 2015).

The Chinese version of the AVLT has multiple versions, such as the Shanghai Mental Health Center version and the Chinese University of Hong Kong version, among which the Huashan Hospital version is widely used in cognitive assessment-related studies in China and has been confirmed to have good reliability and validity (Guo et al., 2007; Zhao et al., 2015). However, our research team found in the study that AVLT is difficult for elderly patients with complications, such as a low level of education, various dialects, and hearing impairment, which predispose subjects to give up halfway through the test. Therefore, we intend to develop a memory assessment scale with better applicability. The brief cognitive ability test (Erzigkeit’s short cognitive performance test), also known as the Syndrom Kurztest (SKT), a cognitive assessment composite scale developed by the German researcher Erzigkeit H, is widely promoted internationally (Choi et al., 2004; Flaks et al., 2009) and is also recommended for POCD assessment (Rundshagen, 2014). The SKT consists of 9 subtests, of which subtests 1, 2, 8, and 9 are part of the memory test (SMOT), which uses cartoon-style pictures containing 12 objects, and the SMOT is less affected by factors such as cultural differences and level of education than AVLT (Choi et al., 2004; Flaks et al., 2009; Rundshagen, 2014). Based on the above background, our research team developed a modified version of the SMOT according to the SMOT picture memory evaluation method and AVLT scoring method, and this study mainly explored its applicability in the elderly population with a low educational level in China (Lu et al., 2021).

## 2. Subjects and methods

### 2.1. Subjects

This study was approved by the Medical Ethics Committee of our hospital and was a cross-sectional observational study. Inclusion criteria were as follows: patients ages 55 to 75 undergoing elective herniorrhaphy in our hospital from 1 March 2019 to 31 October 2021, their accompanying family members, and a willingness to sign informed consent. Exclusion criteria were as follows: inability to communicate effectively in Mandarin; inability to undergo spinal anesthesia due to objective or subjective factors; American Society of Anesthesiologists (ASA) ≥ III; history of central nervous system disease or mental illness; history of malignant tumors; severe chronic diseases (severe heart disease, lung disease, chronic neuralgia and other diseases, or disabilities affecting the quality of life); Mini-Mental State Examination (MMSE) score < 20 points; patients who did not receive spinal anesthesia on the day of surgery; operation times of more than 2 h; serious complications (intraoperative hemodynamic instability, postoperative vomiting, headache, insomnia and delirium); any subjective or objective factors that interrupted the test.

The included subjects were randomly divided into the SMOT and AVLT groups. The SMOT group received the SMOT test twice on the day before surgery and the second day after the operation, while the AVLT group received the AVLT test at the same time point. Randomization method: An on-site lottery was performed in the consultation room before anesthesia, and randomization was performed by using the Excel function “Randbetween (1, 2).” If the value was “1,” the patient was included in the SMOT group, and the accompanying family member was included in the AVLT group; if the value was “2,” the reverse was true. If there were no accompanying family members, only the patient was randomized. The evaluation site was the consultation room before anesthesia, and the time limit for surgery was 15:00–18:00. For the convenience of the study, the evaluators included two young male doctors who could skillfully use the MMSE scale, modified SMOT and AVLT. Evaluator A assesses the patient, while evaluator B assesses the patient’s family member. To ensure the consistency and proficiency of the two assessors in the operation of the guided language of the cognitive assessment measurement form, the two assessors successively pretested the MMSE scale and the SMOT and AVLT immediate recall test on more than 30 volunteers (ages 55–75) before the study, and the two assessors were present at the same time to learn from each other during the evaluation.

### 2.2. Tool

#### 2.2.1. AVLT operating process

The subjects were told in advance that they would be asked to recall words. The evaluator then read 12 words a second apart. After reading, the subject was required to recall immediately, and the test was conducted three times. The average of the correct words recalled three times was recorded as the “immediate recall” score; 20 min later, the subject was required to recall the words again, and the correct number of words recalled was recorded as the “delayed recall” score. During the 20-min interval, all subjects performed two fixed non-verbal tests, as did the SMOT group.

#### 2.2.2. SMOT improved method and operation process

According to Chinese cultural characteristics, the original cartoon style and picture colors were maintained in the 12 types of object pictures; unlike the original presentation, in our study, the 12 pictures were presented to the subjects in turn rather than altogether. As with the AVLT procedure, subjects were told in advance that they would be asked to recall the pictures. The assessor then presented 12 pictures to each subject in a fixed order and asked the subject to name each picture. Each picture was separated by 1 s, and the exercise was repeated three times. It was not necessary for the subject to give the accurate name of the object shown in the picture; for example, he or she could call a chair a stool, as long as it was evident that the subject understood what the object was. If a subject was unable to name the object immediately, the rater would explain it, and if the subject was still unable to recognize the object during the next two exercises, the rater would explain it again but would not score the picture for either immediate or delayed recall.

### 2.3. Anesthesia methods and management of surgical patients

Routine ECG monitoring was performed after admission, and sodium lactate Ringer’s injection (6 ml/kg) was infused in advance after opening the upper limb venous access. The L3-4 or L2-3 interspace was selected at the puncture site for spinal anesthesia. After the cerebrospinal fluid reflux was unobstructed, 2∼3 ml of heavy 0.5% bupivacaine was slowly injected, and the anesthesia level was adjusted to the T10 level. No sedative drugs were used during the operation, and the patient was asked to go to the pillow supine position for 6 h after the operation.

### 2.4. Statistical analysis

SPSS 20.0 software was used for statistical analysis. Measurement data were expressed as the mean ± standard deviation, and enumeration data were expressed as the number of cases and/or rate (%). Independent sample *t*-test, paired sample *t*-test, Chi-square test, multiple linear regression analysis and repeated measures analysis of variance were used for statistical methods. See the section “Results” for specific methods. *P* < 0.05 was considered statistically significant.

## 3. Results

### 3.1. Randomization of the study

After screening for inclusion and exclusion criteria, a total of 312 subjects were randomized; after removal, the data of a total of 228 subjects were included in the analysis: the SMOT group (*n* = 121) and AVLT group (*n* = 107), of which the proportion of AVLT that did not finish the test was significantly higher than that of SMOT (χ^2^ = 10.513, *P* = 0.002). And the reasons for excluding 84 cases included 36 cases with operation time over 2 h, 28 cases without epidural anesthesia on the day of operation, 8 cases with postoperative pain, and 12 cases with postoperative nausea.

### 3.2. Comparison between patients and their accompanying family members

There were no significant differences in general data, such as age, sex, education, and two MMSE scores, between surgical patients and their families set as controls except for ASA grade (Table 1).

**TABLE 1:** Comparison of general data between patients and their families.

Measures	Patients (*n* = 132)	Controls (*n* = 96)	Statistics	*P*-value
Age, year	63.9 ± 5.3	64.6 ± 5.3	*t* = –1.013	0.311
Sex, female/male	73/59	43/53	χ^2^ = 3.856	0.060
Education ≤ primary school/ ≥ junior high school^a^	67/65	51/45	χ^2^ = 0.377	0.576
Occupations, category A/category B^b^	64/68	53/43	χ^2^ = 0.721	0.423
ASA, I/II	56/76	54/42	χ^2^ = 4.249	0.041*
Preoperative MMSE score	24.8 ± 2.2	24.6 ± 2.2	*t* = 0.558	0.574
Postoperative MMSE score	26.0 ± 2.4	26.0 ± 2.3	*t* = –0.240	0.808

^a^Because the proportion of illiterate subjects and those with a high school degree or more was too low, the education level is only divided into two levels: primary school degree or less; junior high school degree or more.

^b^Occupations are classified into two categories according to whether the daily work (before retirement) involved reading, writing, or computer operation: Category A was composed of teachers, doctors, civil servants, etc.; Category B, farmers, housework, caregivers, drivers, etc.

*p < 0.05.

### 3.3. General data comparison of the SMOT AVLT group

There was no significant difference in age, sex, identity, education, or other general data between the two groups (Table 2).

**TABLE 2:** General data comparison of the SMOT AVLT group.

Measures	Group SMOT (*n* = 121)	Group AVLT (*n* = 107)	Statistics	*P*-value
Age, year	63.9 ± 5.7	64.8 ± 5.2	*t* = –0.818	0.411
Sex, female/male	65/56	48/59	χ^2^ = 1.513	0.246
Patients/accompanying family members	71/50	64/43	χ^2^ = 0.000	1.000
≤ Primary school/ ≥ junior high school	67/54	53/54	χ^2^ = 1.363	0.277
Occupations, category A/category B	61/60	55/52	χ^2^ = 0.169	0.687
ASA, I/II	59/62	55/52	χ^2^ = 0.016	1.000
Preoperative MMSE score	24.8 ± 2.1	24.6 ± 2.3	*t* = 0.524	0.600

### 3.4. Comparison of SMOT and AVLT first scores

Repeated measures analysis of variance showed that there was a significant difference in the three learning scores between the SMOT and AVLT (*F* = 19.249, *P* = 0.000), so multivariate analysis of variance was used, and the results showed that the three learning scores of the SMOT were significantly higher than those of the AVLT. An independent sample *t*-test showed that the immediate recall and delayed recall scores of the SMOT were significantly higher than those of the AVLT (Table 3).

**TABLE 3:** Comparison of the SMOT to AVLT first scores.

Measures	First learn	Second learn	Third learn	Immediate recall	Delayed recall
SMOT (*n* = 121)	5.6 ± 1.7	7.3 ± 2.0	8.6 ± 1.8	21.9 ± 5.0	6.4 ± 1.8
AVLT (*n* = 107)	4.8 ± 1.8	6.6 ± 2.1	7.5 ± 2.1	18.8 ± 5.3	5.6 ± 2.4
Statistics	*F* = 12.659	*F* = 10.980	*F* = 22.226	*t* = 4.375	*t* = 4.055
*p*-value	0.000*	0.001*	0.000*	0.000*	0.000*

*p < 0.05.

Immediate recall and delayed recall were similar difficulty in both tests (both close to 0.5), and discrimination was also good (both greater than 0.3) (Table 4). According to the discrimination calculation principle, 27% of scores below the total score were defined as low score, and 73% of scores above the total score were defined as high score; that is, low score for immediate recall was ≤ 9 points and high score was ≥ 27 points; low score for delayed recall was ≤ 3 points and high score was ≥ 9 points. The chi-square test showed that the low scores of immediate recall and delayed recall in AVLT were significantly higher than those in SMOT, while the high scores were not significantly different (Table 4).

**TABLE 4:** Difficulties, discrimination, and scoring rates between the SMOT and AVLT.

Measures	Immediate recall				Delayed recall			
	Difficulty	Discrimination	Low score end	High score end	Difficulty	Discrimination	Low score end	High score end
SMOT	0.61	0.28	4 (3.3%)	12 (9.9%)	0.57	0.32	7 (5.7%)	10 (8.2%)
AVLT	0.53	0.39	13 (12.1%)	8 (7.4%)	0.46	0.47	21 (19.6%)	8 (7.4%)
Statistics	–	–	χ^2^ = 8.141	χ^2^ = 0.856	–	–	χ^2^ = 11.158	χ^2^ = 0.049
*p*-value	–	–	0.007*	0.471	–	–	0.001*	1.000

Difficulty, that is, the average score rate, is the mean score of a test divided by its full score; after sorting the test scores from high to low, 27% of the data are selected from the two ends to calculate the average of the two ends, respectively (Forrest et al., 1994), and the discrimination is the average score rate of the high score end minus the average score rate of the low score end.

*p < 0.05.

### 3.5. Analysis of test deviation between SMOT and AVLT

Multiple linear regression analysis with the stepwise method using α = 0.05 and β = 0.1 as dependent variables, age, identity (patient/family member), gender, education, occupation, presence, or absence of media exposure habits, and MMSE basic score as independent variables was performed, and the results showed that age was a significant factor influencing the scores of each test; except SMOT delayed recall, the other tests were closely related to MMSE basic scores; the influencing factors of AVLT test scores were more than SKT tests (Table 5). In addition, there was no significant relationship between the scores of each item of the two tests and the identity of the subjects, further confirming the homogeneity between the family members and the patients.

**TABLE 5:** Analysis of test deviation between SMOT and AVLT.

Measures/Results		Age	Identity	Sex	Education	Occupation	Media exposure habits	MMSE
SKT immediate recall	*B*-value	-0.283	-0.669	-0.140	-0.509	0.723	-0.176	0.588
	*p*-value	0.000*	0.507	0.876	0.543	0.411	0.841	0.003*
AVLT immediate recall	*B*-value	-0.314	0.078	-0.737	1.780	-2.995	-0.032	0.886
	*p*-value	0.000*	0.913	0.360	0.025*	0.001*	0.982	0.000*
SKT delayed recall	*B*-value	-0.106	0.108	-0.182	0.017	0.407	-0.308	0.091
	*p*-value	0.000*	0.730	0.554	0.936	0.173	0.319	0.149
AVLT delayed recall	*B*-value	-0.153	-0.283	-0.451	0.313	-0.757	-0.231	0.218
	*p*-value	0.000*	0.435	0.183	0.359	0.041*	0.551	0.011*

*p < 0.05.

### 3.6. Within-group comparison of SMOT and AVLT scores

A paired *t*-test showed that the SMOT second immediate recall score was significantly higher than its first, and there were no significant differences in the two scores on the remaining tests; the high score rate of the SMOT second immediate recall and delayed recall was significantly higher than the first, and there was no significant difference in the scores of the remaining tests (Table 6). In addition, an independent sample *t*-test showed that there were no significant differences in scores between family members and patients in the second test, once again confirming the homogeneity of the two.

**TABLE 6:** Within-group comparison of SMOT and AVLT scores.

Measures		SMOT					AVLT				
		Average score	Difficulty	Discrimination	Low score rate	High score rate	Average score	Difficulty	Discrimination	Low score rate	High score rate
Immediate recall		23.1 ± 5.1	0.65	0.37	2/121	24/121	19.0 ± 6.0	0.53	0.42	10/107	14/107
vs. Preoperative	Statistics	*t* = –3.967	–	–	χ^2^ = 0.000	χ^2^ = 4.669	*t* = –0.829	–	–	χ^2^ = 0.443	χ^2^ = 3.248
	*p*-value	0.000*	–		1.000	0.046	0.413	–	–	0.660	0.117
Delayed recall		6.9 ± 1.8	0.57	0.38	1/121	26/121	5.9 ± 2.2	0.49	0.47	17/107	19/107
vs. Preoperative	Statistics	*t* = –1.387	–	–	χ^2^ = 4.649	χ^2^ = 7.523	*t* = –1.763	–	–	χ^2^ = 0.491	χ^2^ = 3.671
	*p*-value	0.157	–	–	0.061	0.010*	0.088	–	–	0.608	0.079

*p < 0.05.

## 4. Discussion

The consensus of multiple international POCD clinical research teams, including ISPOCD, is that multiple neuropsychological test tools must be used in combination for the assessment and diagnosis of POCD (Rasmussen et al., 2001; Hanning, 2005; Rundshagen, 2014). In contrast, in recent years, few researchers have used neuropsychological test batteries in domestic POCD clinical research, and most research teams tend to use comprehensive cognitive assessment scales, of which the MMSE scale is the most widely used (Xiao et al., 2017; Zhang et al., 2017). The MMSE scale is mostly used for dementia screening, and for mild cognitive impairment; its sensitivity and specificity are poor, so most researchers believe that it is not suitable for assessing POCD (Rasmussen et al., 2001; Hanning, 2005; Lin et al., 2013; Rundshagen, 2014). This study also found that there were many problems in the MMSE scale as follows: (1) the difficulty of the first evaluation of MMSE in all subjects was 0.83, and the discrimination was 0.16; (2) the learning effect was significant, and the paired *t*-test showed that the second MMSE score in all subjects (26.0 ± 2.4) was significantly higher than the first score (24.7 ± 2.2) (*t* = –15.114, *P* = 0.000); (3) there may be a ceiling effect in the qualitative ability and language ability tests; (4) there may be a floor effect in the attention and calculation tests for subjects with low education levels; (5) the memory evaluation part contained only three words, with too low sensitivity. Therefore, our research team intends to develop a set of neuropsychological test batteries suitable for evaluating POCD in middle-aged and elderly Chinese patients, and this study is one of the research topics in this research direction.

The reasons for selecting patients undergoing elective herniorrhaphy as the observation subjects in this study are as follows: (1) compared with recruiting volunteers, surgical patients are more convenient for follow-up and greatly reduce the loss rate. The total loss rate in this study was 15.1%. All subjects were accompanied by family members. Surgical patients are more likely to cooperate and not likely to give up on taking the test. The 43 subjects who did not complete the test were accompanied by 32 family members. (2) Compared with patients undergoing major surgery and medical inpatients, such patients undergoing elective minor surgery are about the same as the normal population, and this study was strictly limited in the inclusion and exclusion criteria. (3) Based on the purpose of the POCD study, it is convenient to observe the specific factors that may affect cognitive evaluation in surgical patients. For example, this study found that intravenous indwelling has a certain effect on a non-verbal test. The reasons for including family members as subjects in this study are : (1) to facilitate follow-up; (2) expand the sample size to facilitate the study; and (3) serve as a control group to rule out the possible effects of diseases, the inpatient environment, medical intervention and other factors on patients. Study data analysis also confirmed that surgical patients and family volunteers have “homogeneity” in most aspects, especially multiple verifications of cognitive ability. However, there are also some differences, such as the greater proportion of ASA II patients compared to family members, which may be because the medical records of patients are perfect and ASA classification is convenient, while there are more uncertainties in asking the medical history of family members. In addition, although there was no significant difference in the sex ratio between the two groups, there were more males in the family group, which may be attributed to the fact that female patients are mostly accompanied by male family members, while male patients can sign the anesthesia informed consent by themselves.

AVLT is one of the three neuropsychological test methods recommended by the 1995 Consensus Conference on Cognitive Impairment Assessment after Cardiac Surgery (Murkin et al., 1995; Xiao et al., 2017; Zhang et al., 2017) and is also used by many international POCD clinical research teams in addition to ISPOCD (Silbert et al., 2014, 2015). In this study, we used the Huashan version prepared by Professor Guo Qihao, which has confirmed the validity and reliability of AVLT in research fields such as mild cognitive impairment (MCI) (Guo et al., 2007; Zhao et al., 2015) and is used by many researchers in China (Liu et al., 2012; Li et al., 2016). Since our team found that AVLT poses many problems for the elderly population with a low level of education level, we developed a modified version of the SMOT based on the SKT memory subtest. To prepare the SMOT, we designed a total of 20 pictures. After data analysis of 50 subjects aged 55–75 years, the 8 pictures with the lowest identification were removed, and the remaining 12 pictures had 100% identification. In this study, all subjects said the correct name of each object. In this study, we found that the scores of all items of SMOT were significantly higher than those of AVLT, and their difficulty and discrimination were not significantly different from those of AVLT. In addition, according to the principle of discrimination, this study defined “low score” and “high score” to analyze the potential ceiling and floor effects of the test, and the results showed that there was no significant difference in the high score rate between the two tests, but the low score of immediate recall and delayed recall of AVLT was significantly lower than that of SMOT. Further analysis found that approximately 80% of the low score of AVLT was still the low score in the second test, indicating that AVLT was likely to have floor effect on this part of subjects. The test deviation analysis showed that the influencing factors of AVLT were greater than those of SMOT; that is, AVLT had poor general applicability to the population aged 50–70 years, and there was interference in the diagnosis of cognitive impairment. For example, occupation was the influencing factor of AVLT. If there were differences in the proportion of normal occupations, errors would occur when the Z score was calculated with the mean and standard deviation of the normal group to offset the learning effect in the diagnosis of POCD by the *Z*-score. However, the within-group comparison revealed that SMOT had a significant learning effect on immediate recall, and although the test interval was 2 days in this study, although AVLT had no significant learning effect, the low score rate on the second test was still high, and 17 of the 18 patients with low scores on delayed recall also had low scores on the first test, further indicating that AVLT may have a floor effect (Beier et al., 2019; Holmgaard et al., 2019; McGovern et al., 2019).

The subjects observed in this study were middle-aged and elderly populations aged 55–75 years. Because this age group has a high degree of cooperation with difficult neuropsychological tests and has better clinical preventive significance for POCD, it is also the main research target population of our POCD clinical research team. However, from the perspective of norms, the number of observations in this study is still small, and there are regional restrictions. In addition, although the SMOT and AVLT are objective neuropsychological tests, the rater reliability analysis is conducive to ruling out the interference of assessor-related factors. However, this study did not analyze them but made sufficient pretest preparation to ensure the consistency of assessor reliability.

## Data availability statement

The original contributions presented in this study are included in the article/Supplementary material, further inquiries can be directed to the corresponding author.

## Ethics statement

Written informed consent was obtained from the individual(s) for the publication of any potentially identifiable images or data included in this article.

## Author contributions

LC contributed to write the article, critical revision of the article, and statistical analysis. BG performed critical revision of the article, final approval of the article, and statistical analysis. CY performed the data collection and critical revision of the article. HJ performed conception and design, wrote the article, and critical revision of the article. ZW helped perform the data collection and critical revision of the article. All authors contributed to the article and approved the submitted version.
